# Supplementary material for: Synthetic band-structure engineering in polariton crystals with non-Hermitian topological phases
Source: Nat Commun. 2020 Sep 4;11:4431. doi: 10.1038/s41467-020-18213-1 (PMC7474071; doi:10.1038/s41467-020-18213-1)
Supplement: Supplementary file 2 — Supplementary Information [file 41467_2020_18213_MOESM2_ESM.pdf]

Supplementary Information for: Synthetic band-structure engineering in polariton crystals with non-Hermitian topological phases

L. Pickup, H. Sigurdsson, J. Ruostekoski, and P. G. Lagoudakis

CONTENTS

Supplementary Note 1. Driven-dissipative Gross-Pitaevskii theory 2

1.1. Time resolved dispersion 3

1.2. dGPE results 4

Supplementary Note 2. Bloch theorem 7

2.1. Condensation in high-symmetry points 7

2.2. First principle calculation of the Zak phase 8

2.3. Complex Su-Schrieffer-Heeger model of gain-localised polaritons 10

Supplementary Note 3. Simulations of defect condensation 15

Supplementary References 17

Supplementary Note 1. DRIVEN-DISSIPATIVE GROSS-PITAEVSKII THEORY

The transition from a thermally stochastic state to a macroscopic coherent polariton condensate can be captured within the mean field theory approach. The condensate order parameter $\Psi(\mathbf{r}, t)$ is then described by a two-dimensional (2D) semiclassical wave equation referred as the driven-dissipative Gross-Pitaevskii equation (dGPE) coupled with an excitonic reservoir which feeds non-condensed particles to the condensate [1]. The reservoir is divided into two parts: An active reservoir $n_A(\mathbf{r}, t)$ belonging to excitons which experience bosonic stimulated scattering into the condensate, and an inactive reservoir $n_I(\mathbf{r}, t)$ which sustains the active reservoir [2, 3].

$$i\frac{d\Psi}{dt} = \left[-\frac{\hbar\nabla^2}{2m} + g(n_A + n_I) + \alpha|\Psi|^2 + \frac{i}{2}\left(Rn_A - \gamma\right)\right]\Psi, \tag{1a}$$

$$\frac{dn_A}{dt} = -\left(\Gamma_A + R|\Psi|^2\right)n_A + Wn_I, \tag{1b}$$

$$\frac{dn_I}{dt} = -\left(\Gamma_I + W\right)n_I + P(\mathbf{r}). \tag{1c}$$

Here, m is the effective mass of a polariton in the lower dispersion branch, α is the interaction strength of two polaritons in the condensate, g is the polariton-reservoir interaction strength, R is the rate of stimulated scattering of polaritons into the condensate from the active

reservoir, γ is the polariton condensate decay rate, $\Gamma_{A,I}$ are the decay rates of active and inactive reservoir excitons respectively, W is the conversion rate between inactive and active reservoir excitons, and $P(\mathbf{r})$ is the non-resonant continuous wave pump profile. The polariton mass and lifetime are based on the sample properties: $m = 0.32$ meV ps^2 μm^{-2} and $\gamma = 1/5.5$ ps^{-1}. We choose values of interaction strengths typical of GaAs based systems: $\hbar\alpha = 1.6$ μeV μm^2 and $g = 2\alpha$. The non-radiative recombination rate of inactive reservoir excitons is taken here much smaller than the condensate decay rate $\Gamma_I^{-1} = 500$ ps whereas the active reservoir is taken comparable to the condensate lifetime $\Gamma_A^{-1} = 5$ ps due to fast thermalisation to the exciton background [4] and partly because active excitons are within the light cone. The final two parameters are then found by fitting numerical results to experiment. This gives the values $\hbar R = 36.3$ μeV μm^{-2}, and $W = 0.05$ ps^{-1}.

The pump is written,

$$P(\mathbf{r}) = P_0 f(\mathbf{r}) = P_0 \sum_{n=1}^{N} \exp\left[-(x^2 + y_n^2)/2w^2\right]. \tag{2}$$

where N is the number of pumps in question and y_n their coordinates along the y-axis, P_0 denotes the pump power density and w corresponds to a 2 μm full-width-half-maximum (FWHM).

1.1. Time resolved dispersion

Here we discuss a method to numerically time-resolve the dispersion of the Schrödinger equation in some arbitrary potential $V(\mathbf{r})$. The linear parts of the dGPE form a non-Hermitian Schrödinger equation written,

$$i\frac{d\Psi}{dt} = \left[-\frac{\hbar\nabla^2}{2m} + g(n_A + n_I) + \frac{i}{2}\left(Rn_A - \gamma\right)\right]\Psi. \tag{3}$$

For nonzero pump powers $P_0 \neq 0$, and in the linear regime ($|\Psi|^2 \simeq 0$), the reservoirs converge to a steady state solution satisfying $\dot{n}_{A,I} = 0$. We can then write an effective potential,

$$V(\mathbf{r})/\hbar = g(n_A + n_I) + \frac{iRn_A}{2} = \left[g\left(1 + \frac{W}{\Gamma_A}\right) + \frac{iR}{2}\right]\frac{P(\mathbf{r})}{\Gamma_I + W}. \tag{4}$$

For brevity we will write $V(\mathbf{r}) = V_0 f(\mathbf{r})$ where V_0 is defined,

$$V_0 = \hbar\left[g\left(1 + \frac{W}{\Gamma_A}\right) + \frac{iR}{2}\right]\frac{P_0}{\Gamma_I + W}. \tag{5}$$

Supplementary Equation (3) then becomes

$$i\hbar\frac{d\Psi}{dt} = \left[-\frac{\hbar^2\nabla^2}{2m} + V(\mathbf{r}) - \frac{i\hbar\gamma}{2}\right]\Psi. \tag{6}$$

The dispersion of Supplementary Equation (6) can be extracted by two means. Firstly, by introducing a white noise term $d\theta$ based on a quantum Monte Carlo technique in the Wigner representation [5],

$$i\hbar\frac{d\Psi}{dt} = \left[-\frac{\hbar^2\nabla^2}{2m} + V(\mathbf{r}) - \frac{i\hbar\gamma}{2}\right]\Psi + \frac{d\theta}{dt}, \tag{7}$$

where the white noise correlation functions are written $\langle d\theta(x_i)d\theta(x_j)\rangle = 0$ and $\langle d\theta^*(x_i)d\theta(x_j)\rangle = 2\delta(x_i - x_j)dt$. The dynamics of many random fields generated by $d\theta$ can then be averaged over in k-space (k_x-k_y plane) to produce the dispersion experienced by particles in the system. This method is particularly useful for potentials which lack the usual symmetries required by Bloch theorem to work. Alternatively, if the system possesses translational symmetry then Bloch's theorem can be applied (see Supplementary Note 2).

1.2. dGPE results

Comparison between experiment and simulation is shown in Supplementary Figure 1. The upper row shows experiment and lower row the simulation. Supplementary Figure 1(a,b,c) show the condensate real-space, k-space, and dispersion of the photoluminescence (PL) from a chain of $N = 8$ pump spots with uniform spacing between them, $a_l = a_s = 12.4$ µm. Supplementary Figure 1(d,e,f) show the same for a chain of $N = 8$ pumps with two interchanging separation distances between the pump spots, $a_l = 12.4$ µm and $a_s = 8.8$ µm. In Supplementary Figure 1(g,h) we plot the simulated steady state of the condensate order parameter showing the real-space density $|\Psi(\mathbf{r})|^2$ and k-space density $|\hat{\Psi}(\mathbf{k})|^2$ respectively for the uniformly $N = 8$ spaced pump spots, $a_l = a_s = 12.4$ µm. In Supplementary Figure 1(j,k) we plot the simulated steady state of the condensate order parameter showing the real-space density $|\Psi(\mathbf{r})|^2$ and k-space density $|\hat{\Psi}(\mathbf{k})|^2$ respectively for the $N = 8$ pumps with two interchanging separation distances, $a_l = 12.4$ µm and $a_s = 8.8$ µm. Supplementary Figure 1(i & l) show the numerically time-resolved single-particle dispersion (see Supplementary Note 1.1) for the corresponding $N = 8$ uniform and dimerised chains of Gaussian potentials, $V(\mathbf{r}) = V_0 f(\mathbf{r})$, as given by Supplementary Equation (2). Both dispersions are numerically obtained for $V_0 = 1.44$ meV and $\gamma = 0$ in order to avoid band-broadening and to show

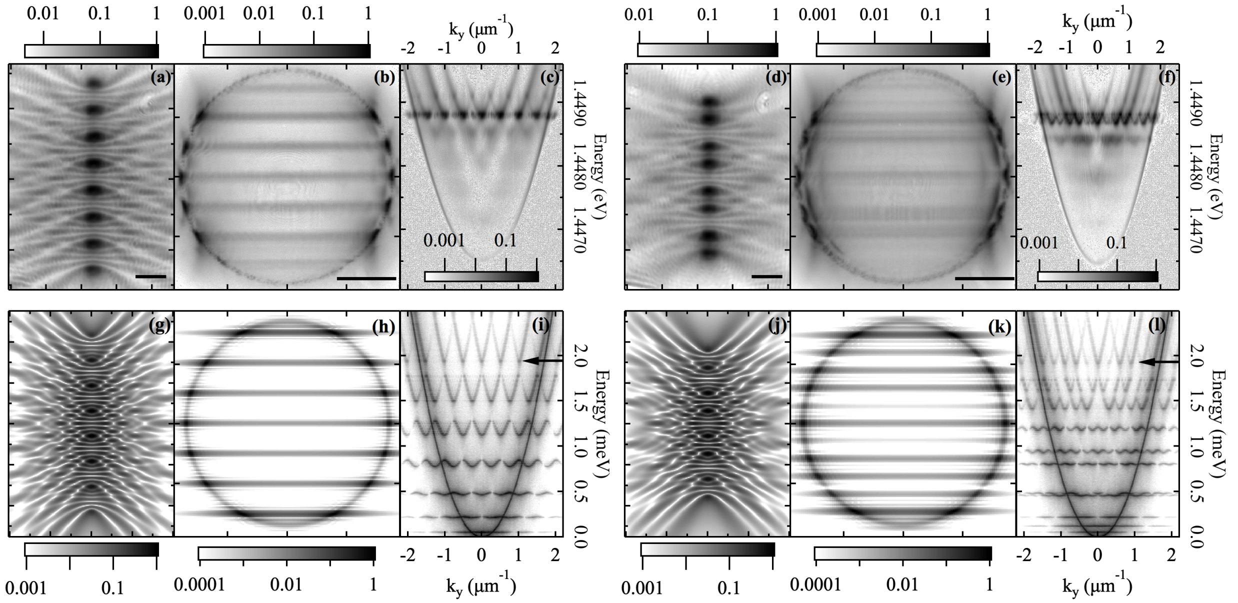

Supplementary Figure 1. Experimental data and the corresponding numerical simulations of uniform and staggered linear chains of polariton condensates. (a,d) Experimental PL from a chain of eight polariton condensates in real space, (b,e) k-space, and (c,f) the dispersion. The lattice constant is $a = 13$ µm in (a,b,c) and alternating with $a_s = 8.6$ µm and $a_l = 13$ µm in (d,e,f). The solid lines on the bottom right of (a,d) represents 15 µm and in (b,e) represents 1 µm^{-1}. In the lower row we show the corresponding steady state condensate wavefunction obtained from simulating Supplementary Equation (1) in (g,j) real space and (h,k) k-space. Panels (i,l) show the time resolved single-particle dispersion of the system for $V_0 = 1.44$ meV and $\gamma = 0$. The energy of the simulated condensates in (g,h) and (j,k) is found to be $E \sim 1.9$ meV which lies in the high-symmetry points of the 7th and 11th bands in their respective dispersions (marked with black arrows). Pump power is set to $P_0 = 19$ ps^{-1} µm^{-2}.

the low energy band structure more clearly. The reason we display only the single-particle dispersion in Supplementary Figure 1(i,l), as opposed to the dispersion containing the frequency components of the condensates, is to show more clearly the bands belonging to all surrounding frequency components which are not part of the condensate. The energy of the simulated condensates in Supplementary Figure 1(g,h) and (j,k) is found to be $E \sim 1.9$ meV which lies in the high-symmetry points of the 7th and 11th band in their respective dispersions (indicated with black arrows).

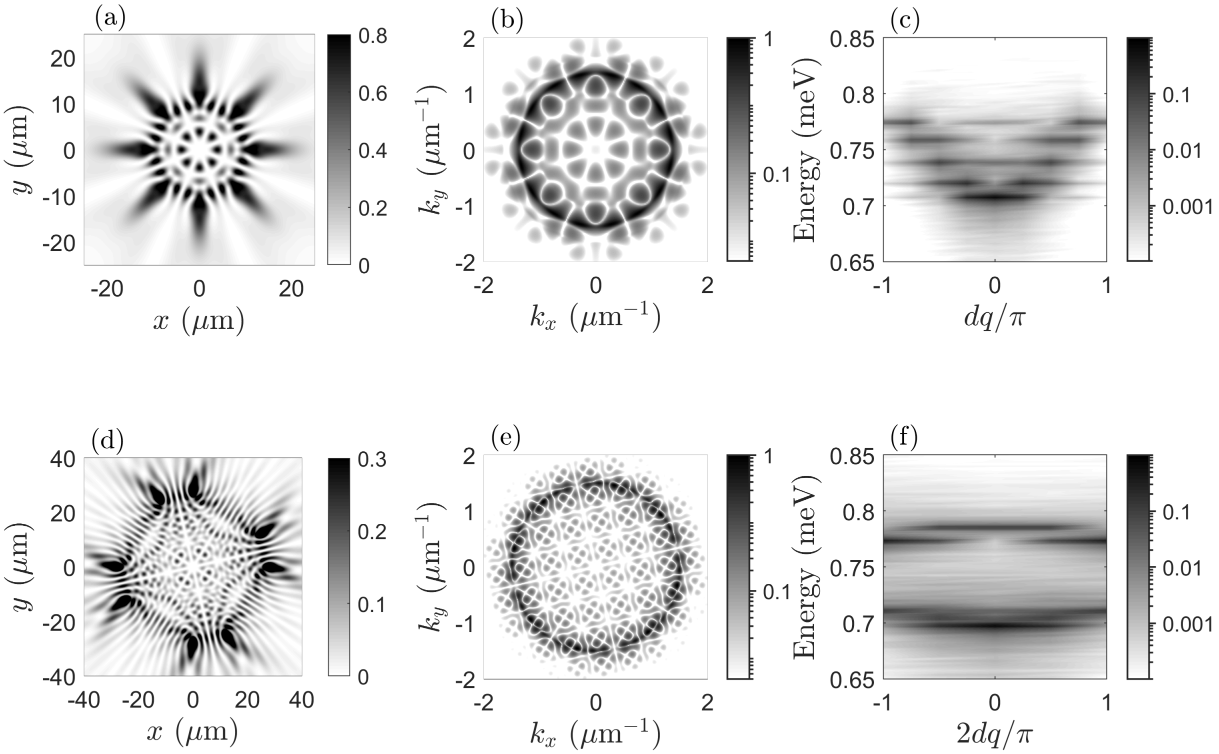

Supplementary Figure 2. Simulation of eight condensates forming a circle for both uniform and staggered separation distance geometries. Simulated steady state condensate (a,d) real-space density $|\Psi(\mathbf{r})|^2$ and (b,e) k-space density $|\hat{\Psi}(\mathbf{k})|^2$ for a regular and dimerised octagon pump pattern respectively. Densities in all panels are normalized to unity. Panels (a,d) are saturated at 80% and 30% respectively to show more clearly the underlying interference pattern. (c,f) Numerically resolved dispersion of a uniform and staggered octagon respectively along its circumcircle showing gap opening of the discrete wavenumbers q corresponding to angular particle propagation.

In Supplementary Figure 2(a,d) we show the real-space and (b,e) k-space densities of the simulated steady state condensate order parameter $\Psi(\mathbf{r})$ for a regular ($P_0 = 15$ ps^{-1} μm^{-2}) and dimerised ($P_0 = 19$ ps^{-1} μm^{-2}) octagon pump pattern respectively. The simulations illustrate the good agreement between the dGPE and the observed experimental PL (see Fig. 6 in main text). In Supplementary Figure 2(c,f) we show an example numerically resolved dispersion of the polygon's angular modes with discrete wavenumber q on the octagon's circumcircle for non-staggered and a staggered scenario respectively. Here, the radius was taken $R = 12.8$ μm and the staggering $\Delta = 0.2$ μm giving approximately $d = 10$ μm distance between potentials for the non-staggered case and $d - \Delta = 9.8$ μm and

$d + \Delta = 10.2$ μm for the staggered case resulting in opening of the gap. Potential strength was taken $V_0 = 1.44$ meV.

Supplementary Note 2. BLOCH THEOREM

Polariton condensation into the high-symmetry points of the Brillouin zone can be understood by scrutinising the linear part of the dGPE [see Supplementary Equation (6)] in the pump lattice bulk by assuming that the system is infinite and periodic such that $V(y) = V(y + L)$ with L being the lattice constant. We can then apply Bloch's theorem where we write the polariton single particle wavefunction in the factorised form of crystal momentum $k_y = q$ along the pump chain and Bloch states in the nth band $u_{n,q}(y)$,

$$\Psi_n(y) = e^{iqy} u_{n,q}(y). \tag{8}$$

Here, $u_{n,q}(y) = u_{n,q}(y + L)$, and the x-coordinate notation is dropped since the system is only periodic along y. Substituting Supplementary Equation (8) into Supplementary Equation (6) the Bloch eigenvalue problem then reads,

$$\left[\frac{\hbar^2}{2m} \left(q - i \frac{\partial}{\partial y} \right)^2 + V(y) - \frac{i\hbar\gamma}{2} \right] u_{n,q}(y) = E_{n,q} u_{n,q}(y). \tag{9}$$

We can diagonalise Supplementary Equation (9) by expressing the Gaussian potential as a Fourier series in the basis of the reciprocal lattice vectors $G = 2\pi/L$ [6],

$$V(y) = \sum_G V_G e^{iGy}. \tag{10}$$

2.1. Condensation in high-symmetry points

The top eight bands $E_{n,q}$ in the Brillouin zone of the system for $V_0 = 1.44 + i0.5$ meV are shown in Supplementary Figure 3. The value of V_0 is extracted from gDPE simulations previously shown in Supplementary Figure 1. The colorscale denotes energies with low imaginary part (blue) and large imaginary part (red). We stress that the imaginary part of the energies $E_{n,q}$ corresponds directly to the gain of the Bloch waves. Energies of highest gain are found in the Γ and M points of the Brillouin zone of positive effective mass, which can be

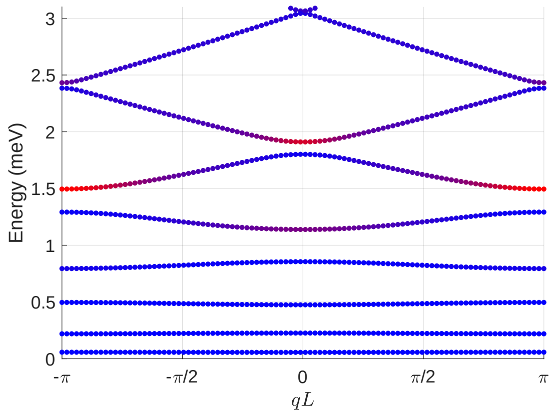

Supplementary Figure 3. Calculated bands using Bloch's theorem for complex valued Gaussian potentials in a uniform lattice with parameters: $L = 12.4$ µm, $w = 0.85$ µm, $V_0 = 1.44 + i0.5$, $m = 0.32$ meV ps^2 µm^{-2} and $\gamma = 1/5.5$ ps^{-1}. The colorscale indicates crystal momenta with low (blue) and high (red) optical gain, i.e. positive imaginary energy.

intuitively understood from the Bloch states in these points having maximum overlap with the gain (pump) region. The fact that bands 6 and 7 possess optimal gain can be understood by inferring that lower energy Bloch waves have poor penetration (overlap) into the repulsive potentials which contain all the gain, whereas higher energy Bloch waves transmit too easily through the gain regions. Therefore, only certain bands will have "ideal" penetration into the gain region of the pump induced potentials. As a consequence, condensation into the crystal bands high-symmetry points can be predicted by straightforward diagonalisation of the Schrödinger equation. A similar observation was reported in Ref. [7] but with anti-phase modulation of the real- and imaginary-parts of the lattice potentials (i.e., there regions of $\text{Re}(V) > 0$ corresponded to $\text{Im}(V) < 0$).

2.2. First principle calculation of the Zak phase

The continuum Bloch model [Supplementary Equation (9)] can be applied to calculate the geometric phase arising in periodic systems. A well known formula for the geometric (Berry) phase accumulated when the wavefunction traverses a closed path in parameter space is written [8],

$$\gamma_C = \oint_C \langle u_{n,q} | \partial_q | u_{n,q} \rangle \, dq, \tag{11}$$

where the integral is taken over a closed path C in the Bloch states parameter space, typically the Brillouin zone's edge. For a one-dimensional (1D) lattice system the geometric phase γ_C is called the Zak phase [9], which we label $\mathcal{Z}$. We will be concerned with the discretisation of the Brillouin zone in small reasonable steps of momentum $q \in \{0, \delta_q, 2\delta_q, \ldots J\delta_q\}$ where J is an integer, and $J\delta_q = 2\pi/L$, and L is the size of the lattice unit cell. We can write a more transparent form to our discretised crystal momentum parameter $q_j = j\delta_q$. A convenient gauge invariant method to numerically calculate the Zak phase of the n-th band takes advantage of the fact that $u_{n,q+\delta_q} \approx u_{n,q} + \partial_q u_{n,q} dq$, and the accumulated phase can then be written,

$$\mathcal{Z} = \mathrm{Im} \log \prod_{j=0}^{J-1} \langle u_{m,q_j} | u_{m,q_{j+1}} \rangle, \tag{12}$$

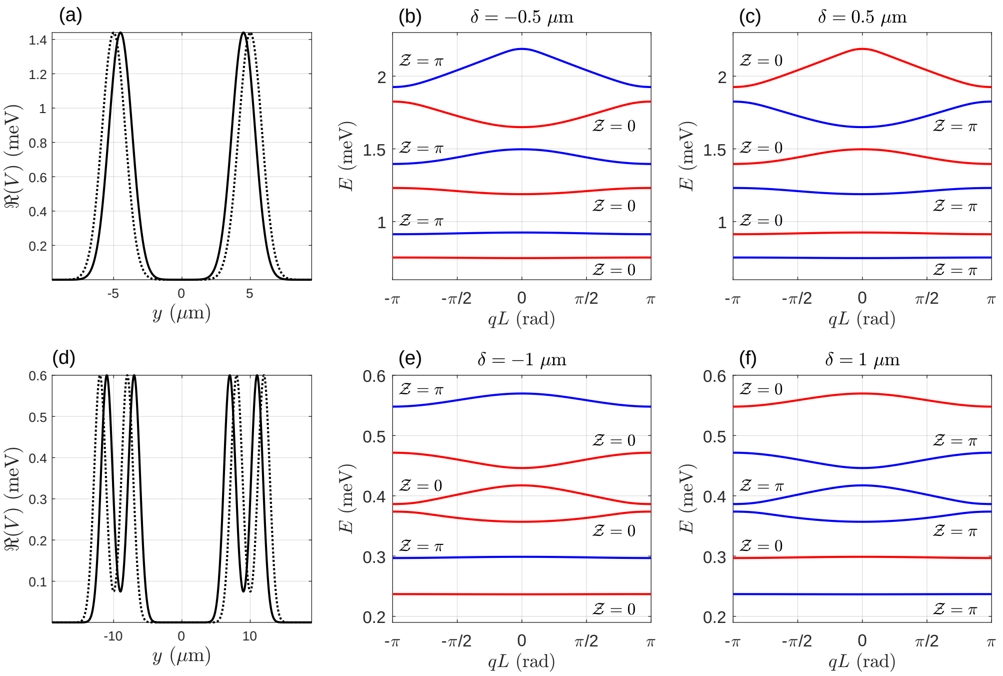

Supplementary Figure 4. Calculated Bloch states and their corresponding Zak phase for the two choices of inversion centre. (a,d) Unit cell, $L = 19$ µm, of the distance staggered (dimerised) potentials under consideration for calculation of Bloch states and their Zak phases. Solid and dotted lines denote the two choices of origin with inversion symmetry corresponding to $\delta = \mp 0.5$. Panels (b,c) show band structure and Zak phase of each band corresponding to potential (a) for the two distinct inversion symmetry points of the lattice. Panels (e,f) show the same calculation but for the potential given in panel (d) and $\delta = \mp 1$.

where it is understood that $u_{m,q_N}(y) = e^{-iGy} u_{m,q_0}(y)$ (see e.g., Refs. [10] and [11]). Results are presented in Supplementary Figure 4 for two separate cases. In Supplementary Figure 4(a,b,c) we scrutinise the case of distance staggered complex Gaussian potentials with two different distances between the Gaussian, $l_{1,2} = d \pm \delta$, with the unit cell size $L = l_1 + l_2 = 2d = 19$ µm. For $\delta = 0.5$ µm we observe each band interchangebly swapping from $\mathcal{Z} = 0$ and π. When $\delta = -0.5$ µm the dimerisation is reversed (roles of l_1 and l_2 are swapped) and all bands undergo a π change in their Zak phases, marking a topological phase transition similar to reports in photonic crystal systems [12]. Here we set $V_0 = 1.44 + i0.5$ meV.

In Supplementary Figure 4(d,e,f) we consider a different geometry which can be accessed with current experimental technology. Consider a pair of Gaussians brought closely together forming a tight confining potential like a tuning fork. Next, consider two pairs of such Gaussian forks which allow polaritons in one fork to interfere with polaritons in the next (see Supplementary Figure 4d). When the distance between the centres of the forks is dimerised the resulting Zak phases [Supplementary Figure 4(e,f)] now display a different order from that of Supplementary Figure 4(b,c). Indeed, the third and the fourth band now form a pair sharing the same Zak phase value. When the dimerisation is reversed, the Zak phase of each band changes by a unit of π marking a topological phase transition. Here we set $V_0 = 0.6 + i0.1$ meV and $L = 19$ µm.

2.3. Complex Su-Schrieffer-Heeger model of gain-localised polaritons

If the system of weakly interacting condensates is discretised then it can be shown that the staggered interference between condensates makes up a non-Hermitian version of the Su-Schrieffer-Heeger (SSH) model [13]. In this section we derive a single band picture of the weakly interacting condensates in the non-Hermitian lattice of Gaussian potentials. Our starting point will be Supplementary Equation (6) considering the interaction between two polariton wavefunctions, gain-localised at their respective potentials, and separated by a distance $|y_1 - y_2| = d$. Since the band physics apply only to the longitudinal axis of the lattice we will treat our problem as a 1D one. The basis of two gain-localised wavefunctions is chosen as,

$$\Psi(y,t) = c_1(t)\phi_1(y) + c_2(t)\phi_2(y), \tag{13}$$

where

$$\phi_n(y) = \sqrt{\kappa} e^{ik|y-y_n|}, \qquad k = k_c + i\kappa. \tag{14}$$

Here $k_c, \kappa > 0$ represents the outflow momentum and decaying envelope of the polaritons generated at each potential. Away from the pump spot the decaying polaritons will adopt an envelope given by $\kappa = m\gamma/2\hbar k_c$ [4]. For typical values from experiment and sample properties, $k_c = 1.7\ \mu\text{m}^{-1}$, $m = 0.32$ meV ps^2 μm^{-2}, and $\gamma = 1/5.5\ \text{ps}^{-1}$, we have $\kappa = 0.026$ μm^{-1}. The rate of change in the wavefunction as a function of the spatial coordinate is then dominated by its momentum k_c, and we can drop κ where appropriate in the following analysis. The small width of the potentials (2 μm FWHM) allows us to treat the envelope of the wavefunction $\Psi(y)$ as slowly varying with the potentials approximately regarded as delta-like scatterers when $2\pi/k_c >$ FWHM.

Taking the inner product over the coordinate basis we have several separate integrals to evaluate in order to find the discretised set of equations,

$$i\hbar \int \Psi(y,t)^* \frac{d\Psi(y,t)}{dt}\, dy = \int \Psi(y,t)^* \left(-\frac{\hbar^2 \partial_y^2}{2m} + V_0 \sum_{n=1}^{N} \delta(y - y_n) - i\frac{\hbar\gamma}{2} \right) \Psi(y,t)\, dy \tag{15}$$

The integrals which arise can be listed as follows:

$$\int_{-\infty}^{\infty} \phi_1^* \left(-\frac{\hbar^2 \partial_y^2}{2m} \phi_1 \right) dy = \frac{\hbar^2}{2m} \left[k^2 - 2ik\kappa \right] \tag{16}$$

$$\begin{aligned} \int_{-\infty}^{\infty} \phi_2^* \left(-\frac{\hbar^2 \partial_y^2}{2m} \phi_1 \right) dy &= -\frac{\hbar^2}{2m} \left[2ik\kappa e^{-ik_c d} - k^2 \left(\cos(k_c d) + \frac{\kappa}{k_c} \sin(k_c d) \right) \right] e^{-\kappa d} \\ &\approx -\frac{\hbar^2}{2m} \left[2ik\kappa e^{-ik_c d} - k^2 \cos(k_c d) \right] e^{-\kappa d} \end{aligned} \tag{17}$$

The approximation is valid for our polariton systems since $\kappa \ll k_c$. The rest of the integrals become,

$$\int_{-\infty}^{\infty} \phi_1^* V(y) \phi_1 dy = V_0 \int_{-\infty}^{\infty} |\phi_1|^2 (\delta(y) + \delta(y-d)) dy = V_0 \kappa (1 + e^{-2\kappa d}), \tag{18}$$

$$\int_{-\infty}^{\infty} \phi_2^* V(y) \phi_1 dy = V_0 \int_{-\infty}^{\infty} \phi_2^* \phi_1 (\delta(y) + \delta(y-d)) dy = 2V_0 \kappa e^{-\kappa d} \cos(k_c d), \tag{19}$$

$$\int_{-\infty}^{\infty} \phi_2^* \phi_1 dx \approx e^{-\kappa d} \cos(k_c d). \tag{20}$$

Supplementary Equation (6) is now in the form of an algebraic differential equation,

$$i\hbar \frac{dc_{1,2}}{dt} + i\hbar e^{-\kappa d} \cos(k_c d) \frac{dc_{2,1}}{dt} = F(c_{1,2}, c_{2,1}) \tag{21}$$

where

$$F(c_1,c_2) = \left(\frac{\hbar^2k^2}{2m} - i\frac{\hbar\gamma_c}{2}\right)\left(c_1 + e^{-\kappa d}\cos{(k_c d)}c_2\right) + \left(\left[V_0 - \frac{i\hbar^2 k}{m}\right]c_1 + V_0 e^{ikd}c_2\right)\kappa$$
$$+\left(\left[V_0 - \frac{i\hbar^2 k}{m}\right]c_2 + V_0 e^{ikd}c_1\right)\kappa e^{-ik^*d}. \tag{22}$$

Since $e^{-\kappa d}\cos{(k_c d)} < 1$ for $0 < d$ the differential-algebraic system of equations can be written as a system of ordinary differential equations. Additionally, assuming the coupling between the wavefunctions is weak, i.e. small $\xi = e^{-\kappa d}$ such that all terms of order $\mathcal{O}(\xi^2)$ and higher can be omitted, we arrive at an expression describing the interference between two spatially separated, gain localized, polariton wavefunctions,

$$i\hbar\frac{dc_{1,2}}{dt} = \left[\frac{\hbar^2(k_c^2+\kappa^2)}{2m} - i\frac{\hbar\gamma}{2} + \kappa V_0\right]c_{1,2} + \kappa\left(V_0\cos{(k_c d)} - \frac{\hbar^2(k_c+i\kappa)}{m}\sin{(k_c d)}\right)e^{-\kappa d}c_{2,1}. \tag{23}$$

Assuming that the momenta k_c and the envelopes κ of the gain-localised polariton wavefunctions are weakly affected by the coupling, the natural frequencies of each condensate is constant, and the correction in energy for the weakly interacting condensates is given by the following normal-mode splitting problem,

$$i\hbar\frac{dc_{1,2}}{dt} \approx \Omega c_{1,2} + \kappa\left(V_0\cos{(k_c d)} - \frac{\hbar^2 k_c}{m}\sin{(k_c d)}\right)e^{-\kappa d}c_{2,1}. \tag{24}$$

Here Ω is a constant denoting the natural frequency of the decoupled wavefunctions. In order to capture the correct distance dependence of the envelope we must replace the exponential term $\kappa e^{-\kappa d}$ from the 1D treatment with the 2D envelope of the Hankel function [4],

$$i\hbar\frac{dc_{1,2}}{dt} = \Omega c_{1,2} + \eta\left(V_0\cos{(k_c d)} - \frac{\hbar^2 k_c}{m}\sin{(k_c d)}\right)|H_0^{(1)}(k_c d)|c_{2,1}. \tag{25}$$

where η is a fitting parameter. In the long wavelength limit where $k_c d \ll 1$ and the Hankel function is replaced by some suitably smooth function, the model assumes the standard tight binding form with $\cos{(k_c d)} \approx 1$, and $\sin{(k_c d)} \approx 0$. Extending the above analysis to a system of multiple coupled gain-localized wavefunctions in a chain we can write,

$$\mathrm{i}\hbar\frac{dc_i}{dt} = \Omega c_i + \sum_{\langle ij\rangle} J(k_c|y_i - y_j|)c_j, \tag{26}$$

where the sum goes over nearest neighbours and the function J captures the coupling,

$$J(k_c|y_i-y_j|) = \eta\left(V_0\cos{(k_c|y_i-y_j|)} - \frac{\hbar^2 k_c}{m}\sin{(k_c|y_i-y_j|)}\right)|H_0^{(1)}(k_c|y_i-y_j|)|. \tag{27}$$

When the chain of pumps are distance staggered so the system is described with a short distance $a_s = d - \delta$ and long distance $a_l = d + \delta$ (dimerised system) then the exchange of energy between polaritons at neighboring sites is described by two non-Hermitian hopping amplitudes $J_\pm$,

$$J_\pm = \eta \left(V_0 \cos\left(k_c[d \pm \delta]\right) - \frac{\hbar^2 k}{\mu} \sin\left(k_c[d \pm \delta]\right) \right) |H_0^{(1)}(k_c[d \pm \delta])|. \tag{28}$$

We note that in the continuum limit the above discretised treatment describes a single polariton crystal band for $|y_n - y_m| = \text{const}$. Supplementary Equation (26) then stays accurate if the polaritons generated by the pumps occupy only a single band well separated from all other bands in the 1D pump lattice. It is then understood that the dimerisation of the lattice and consequent splitting of the aforementioned occupied band can be described using Supplementary Equation (26) when the dimerisation is small (i.e., small changes in $|y_n - y_m| = d \pm \delta$ where $\delta \ll d$). In fact, the validity of Supplementary Equation (26) with hoppings (27) can be given an upper bound by noting that the periodic behaviour of the hoppings cannot correctly predict the gap opening for large δ (i.e., gap will start closing). Let us consider a non-staggered lattice with $k_c = n\pi/d$, corresponding to condensates collected in the momentum-space high symmetry points, and uniform hopping $J_0 = J(k_c d)$. Let us change the distance between half of the pumps such that the lattice is staggered with half of the hoppings given by $J_- = J(k_c[d - \delta])$. The gap size is given by $\Delta E = 2|\text{Re}(J_0 - J_-)|$ which monotonically grows until $\partial_\delta J_- = 0$ where it starts closing again. The range of increasing gap size is then determined by $\delta < k_c^{-1} \tan^{-1}(\hbar^2 k_c / \mu V_0)$. For the experimental parameters of Fig. 2 in the main text we have $\delta < 0.64$ μm as the limit to our tight-binding treatment.

We will focus first on the bulk properties of such a dimerised system where we define $|n, \alpha\rangle$ as the polariton state in unit cell n on sublattice α in a chain of $2N$ pumps with periodic boundary conditions ($|1\rangle = |N + 1\rangle$). The periodic boundary conditions are readily reproduced in a polariton experiment using a ring-shaped chain of pump spots such as shown in Fig. 6 in the main text (and with simulations shown in Supplementary Figure 2). The Hamiltonian describing the single-particle polariton chain system then reads,

$$\mathcal{H} = \Omega \sum_{n=1}^{N} \sum_{\alpha} |n, \alpha\rangle\langle n, \alpha| + J_+ \sum_{n=1}^{N} (|n, B\rangle\langle n, A| + \text{h.c.}) + J_- \sum_{n=1}^{N-1} (|n+1, A\rangle\langle n, B| + \text{h.c.}). \tag{29}$$

This can be diagonalised in a transparent manner by transforming to the basis of crystal

momentum $|q\rangle = N^{-1/2}\sum_{n=1}^{N} e^{inq}|n\rangle$,

$$\mathcal{H}_q = \langle q|\mathcal{H}|q\rangle = \begin{pmatrix} \Omega & J_- + J_+ e^{iq} \\ J_- + J_+ e^{-iq} & \Omega \end{pmatrix}, \qquad q \in \{\delta_q, 2\delta_q, 3\delta_q, \dots\} \tag{30}$$

where $\delta_q = 2\pi/N$. The Bloch solutions belonging to the eigenproblem of Supplementary Equation (30) are,

$$|b^{(\pm)}\rangle = \frac{1}{\sqrt{2}}\begin{pmatrix} \pm 1 \\ e^{i\phi(q)} \end{pmatrix}, \tag{31}$$

where $(\pm)$ denotes the upper (conduction) and lower (valence) band of the system. The Zak phase [9], which can only take values 0 or π (modulo 2π) when the origin is chosen at an inversion center of the system, can be calculated straightforwardly by integration over the Brillouin zone,

$$\mathcal{Z} = -\frac{1}{2}\int_{\mathrm{BZ}} \frac{\partial\phi(q)}{\partial q}\, dq. \tag{32}$$

We point out that the exact details of engineering the dimerised lattice are not relevant in the long-wavelength limit (i.e., wavelengths larger than the distance staggering δ) where interesting physical effects can arise. For instance, it is known that the bands at the edges of the reduced Brillouin zone can be linearised and described, in the continuum limit, with a relativistic Dirac Hamiltonian [14, 15]. The same limit can be argued for polaritons with Bloch momenta $q = \pi/2d$ by discretising the kinetic operator in steps appropriately (asymmetrically) coinciding with the lattice sites n,

$$\partial_x^2 \sum_n \psi_n(x) \rightarrow \sum_n \frac{\psi(n-1) - 2\psi(n) + \psi(n+1)}{(d-\delta)(d+\delta)}. \tag{33}$$

It is understood that for a wave traveling along the chain with momentum $q = \pi/2d$ one has $\psi(n+1) = \psi(n)e^{i\pi/4}$, and therefore $\psi_{n-1} = -\psi_{n+1}$.

$$\sum_n \frac{\psi(n-1) - 2\psi(n) + \psi(n+1)}{(d-\delta)(d+\delta)} = -2\sum_n \frac{\psi(n)}{d^2-\delta^2} \approx -2\sum_n \frac{\psi(n)}{d^2}. \tag{34}$$

For the first order derivative one has,

$$\partial_x \sum_n \psi_n(x) \rightarrow \sum_n \frac{\psi(n+1) - \psi(n-1)}{2d} = \sum_n \frac{\psi(n+1)}{d}. \tag{35}$$

The action of the second order derivative can therefore be approximately interpreted as $\partial_x^2\psi(x) \approx -2i\partial_x\psi(x)/d$ which, in the spinor representation of the two sublattices, is the same result shown in Refs. [14, 15]. More importantly, by engineering a defect in the lattice

(such as shown in Supplementary Figure 2c) we create a kink in the condensate, permitting study of a polaritonic analogue of the Jackiw-Rebbi model. Originally, the model describes a 1D Dirac field coupled to a soliton background field containing a phase kink, resulting in topologically protected edge states and peculiar physics such as a fractional fermion number [16]. In the continuum limit (large lattice) the long wavelength physics of the condensatets can then be described by a (1+1)D relativistic Dirac equation for a polariton sublattice spinor $\chi = (\psi_A, -\psi_B)^T e^{i\pi/4}$, where ψ_A and ψ_B denote polaritons at each sublattice of the dimerised chain,

$$H_D = \int \left(-ic\hbar\chi^\dagger \hat{\sigma}_y \frac{\partial}{\partial x} \chi + \hbar g \varphi \chi^\dagger \hat{\sigma}_x \chi \right) dx. \tag{36}$$

Here $c = d(J_+ + J_-)$, $g = J_+ - J_-$, and φ denotes the soliton profile through the engineered defect in the condensate lattice.

Supplementary Note 3. SIMULATIONS OF DEFECT CONDENSATION

If the translational symmetry of the lattice is broken by introducing a defect into the pump geometry gap states appear between bands belonging to different polariton orbitals/modes. The defect geometry is then found to either favor or disfavor condensation at its location (see Fig. 4 in main text and Supplementary Figure 5). To illustrate this finding we simulate the 1D dGPE which is the same as Supplementary Equation (1) with the exception of,

$$\nabla^2 \to (1 - i\Lambda)\partial_y^2, \tag{37}$$

where Λ is a phenomenological energy damping parameter. The defected scenario is illustrated in Supplementary Figure 5a where $L = 9.6$ µm denotes the lattice constant and Δ the size of the defect. Parameters are taken same as in Supplementary Note 1 with the exception of $\hbar R = 17.8$ µeV µm^{-1}, $\Lambda = 0.1$, and $P_0 = 6$ µm^{-1} ps^{-1}.

Supplementary Figure 5(c,f,i,l) show the numerically time-resolved single-particle dispersion (see Supplementary Note 1.1) for the potential given in Supplementary Figure 5a. The reason we display the single-particle dispersion in Supplementary Figure 1(c,f,i,l), as opposed to the intensity of the frequency components belonging to the condensate, is to show more clearly the band structure of the non-condensed system. Supplementary Figure 5(d,g,j,m) instead show the nonstationary condensate spectra, appearing as a single peak

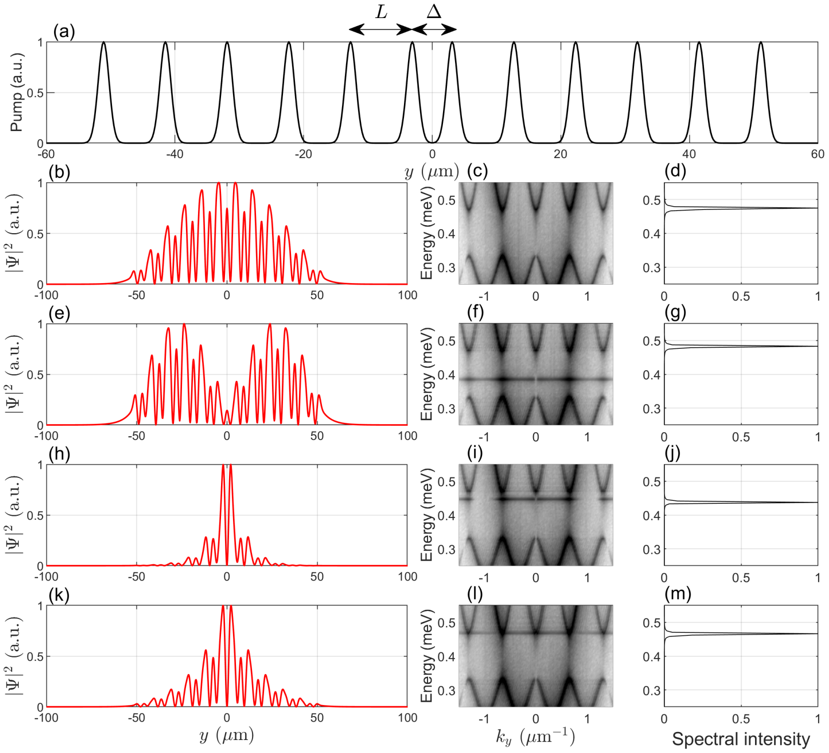

Supplementary Figure 5. Simulation results showing the change in the steady state condensate shape through adjustment of a defect in a uniform 1D lattice. (a) Pump profile $P(y)$ plotted as a function of the lattice longitudinal coordinate illustrating. Here we choose 12 pumps with a lattice constant $L = 9.6$ µm and a defect in the center denoted by distance Δ. Panels (b,e,h,k) show the numerically simulated 1D steady state condensate intensity in real space and corresponding spectral output in (d,g,j,m). Here we have set $\Delta = 9.6$, 8.0, 7.1, 6.2 µm respectively. Panels (c,f,i,l) show corresponding numerically resolved single particle dispersion for clarity with defect state appearing in the bandgap.

which indicates where the condensate would be located in the dispersion in panels (c,f,i,l). For example, in Supplementary Figure 5f we see that the defect mode (dark horizontal line) lies at ~ 0.39 meV, whereas the observed stationary condensate [see Supplementary Figure 5e] occupies a frequency component at ~ 0.48 meV [see Supplementary Figure 5g], which corresponds to frequencies at the bottom of the upper dispersion band instead of the defect mode.

In Supplementary Figure 5b we show the steady state condensate density in the finite

optical polariton crystal for $\Delta/L = 1$. The finite length of the crystal corresponds to open boundaries causing increased losses around the edges. In fact, the additional losses at the crystal edges can be regarded as an effective trapping potential due to the flux of particles from the system [17]. In Supplementary Figure 5e we show the condensate steady state in a crystal with a defect at $\Delta/L = 0.83$ μm corresponding to a flat defect/gap state lifted from the lower band (see dark horizontal line Supplementary Figure 5f). Interestingly, the presence of the gap state causes a sharp drop in condensate real space density even though more power is technically being supplied through the lasers in the middle of the crystal. This seemingly counterintuitive observation is due to the weak evanescent coupling of the defect mode to the rest of the crystal resulting in poor energy flow from the high gain region of the bulk into the defect mode. In Supplementary Figure 5h the defect size is decreased to $\Delta/L = 0.74$ and the condensate energy (see Supplementary Figure 5j) redshifts slightly and now occupies the defect state, consequently becoming highly localised. Lastly, when the defect mode becomes resonant with the upper band at $\Delta/L = 0.65$ (see Supplementary Figure 5l) the condensate in the defect mode couples with the rest of the crystal and becomes more delocalised (see Supplementary Figure 5k). We label these shape preserving nonlinear modes of the condensate in Figs. Supplementary Figure 5e,h as dissipative dark- and bright gap solitons respectively, in analogy with the bright gap solitons observed previously in polariton lattices [18].

We point out that the results shown in Supplementary Figure 5 are stationary solutions of the dGPE. In experiment, we see that this is not always the case where, e.g., in Fig. 4c (main text) the condensate is bimodal, occupying frequencies in both the upper bulk band and the defect state at the same time. In our our calculations in this section, we have focused on the stationary solutions of the dGPE for simplicity but do not exclude the presence of multimodal states coming from the calculation which would depend on the choice of mean field parameters and the details of the potential structure.

SUPPLEMENTARY REFERENCES

[1] Wouters, M. & Carusotto, I. Excitations in a nonequilibrium bose-einstein condensate of exciton polaritons. *Phys. Rev. Lett.* **99**, 140402 (2007).

[2] Lagoudakis, K. G., Pietka, B., Wouters, M., André, R. & Deveaud-Plédran, B. Coherent oscillations in an exciton-polariton josephson junction. *Phys. Rev. Lett.* **105**, 120403 (2010).

[3] Lagoudakis, K. G. *et al.* Probing the dynamics of spontaneous quantum vortices in polariton superfluids. *Phys. Rev. Lett.* **106**, 115301 (2011).

[4] Wouters, M., Carusotto, I. & Ciuti, C. Spatial and spectral shape of inhomogeneous nonequilibrium exciton-polariton condensates. *Phys. Rev. B* **77**, 115340 (2008).

[5] Walls, D. F. & Milburn, G. J. *Quantum Optics* (Springer-Verlag, Berlin Heidelberg, 2008), 2 edn.

[6] Kittel, C. *Introduction to Solid State Physics* (Wiley, New York, 2004).

[7] Nalitov, A. V., Liew, T. C. H., Kavokin, A. V., Altshuler, B. L. & Rubo, Y. G. Spontaneous polariton currents in periodic lateral chains. *Phys. Rev. Lett.* **119**, 067406 (2017).

[8] Wilczek, F. & Shapere, A. *Geometric Phases in Physics* (WORLD SCIENTIFIC, 1989).

[9] Zak, J. Berry's phase for energy bands in solids. *Phys. Rev. Lett.* **62**, 2747–2750 (1989).

[10] Resta, R. Macroscopic polarization in crystalline dielectrics: the geometric phase approach. *Rev. Mod. Phys.* **66**, 899–915 (1994).

[11] King-Smith, R. D. & Vanderbilt, D. Theory of polarization of crystalline solids. *Phys. Rev. B* **47**, 1651–1654 (1993).

[12] Xiao, M., Zhang, Z. Q. & Chan, C. T. Surface impedance and bulk band geometric phases in one-dimensional systems. *Phys. Rev. X* **4**, 021017 (2014).

[13] Su, W. P., Schrieffer, J. R. & Heeger, A. J. Solitons in polyacetylene. *Phys. Rev. Lett.* **42**, 1698–1701 (1979).

[14] Takayama, H., Lin-Liu, Y. R. & Maki, K. Continuum model for solitons in polyacetylene. *Phys. Rev. B* **21**, 2388–2393 (1980).

[15] Ruostekoski, J., Javanainen, J. & Dunne, G. V. Manipulating atoms in an optical lattice: Fractional fermion number and its optical quantum measurement. *Phys. Rev. A* **77**, 013603 (2008).

[16] Jackiw, R. & Rebbi, C. Solitons with fermion number ½. *Phys. Rev. D* **13**, 3398–3409 (1976).

[17] Ostrovskaya, E. A., Abdullaev, J., Fraser, M. D., Desyatnikov, A. S. & Kivshar, Y. S. Self-localization of polariton condensates in periodic potentials. *Phys. Rev. Lett.* **110**, 170407 (2013).

[18] Tanese, D. *et al.* Polariton condensation in solitonic gap states in a one-dimensional periodic

potential. *Nature Communications* **4**, 1749 (2013).
